# Multidomain therapy for Alzheimer’s disease: a scoping review of cognitive decline trials

**DOI:** 10.1186/s13024-025-00912-2

**Published:** 2025-11-22

**Authors:** Jared C. Roach, Gwênlyn Glusman, Molly K. Rapozo, David A. Merrill, Jennifer Bramen, John F. Hodes, Prabha Siddarth, Somayeh Meysami, Shannel H. K. Elhelou, Ryan M. Glatt, Lance Edens, Cory Funk, Dan Kelly, William R. Shankle, Dale Bredesen, Cyrus A. Raji, Leroy Hood

**Affiliations:** 1 Institute for Systems Biology, 401 Terry Ave N, Seattle, WA 98109 USA; 2 Pacific Neuroscience Institute, Santa Monica, CA USA; 3 Department of Translational Neurosciences, Saint John’s Cancer Institute, Santa Monica, CA USA; 4 Pickup Family Neurosciences Institute, Hoag Hospital, Newport Beach, USA; 5 Department of Neurobiology and Behavior, University of California, Irvine, USA; 6 Shankle Clinic, Newport Beach, CA USA; 7 EMBIC Corporation, Newport Beach, CA USA; 8 Departments of Radiology and Neurology, Washington University in St. Louis, St. Louis, MO USA

**Keywords:** Diet, Exercise, Cognitive training, Cognitive decline, Alzheimer’s disease and related disorders (ADRD), Systems biology, Multimodal interventions, Multidomain, Multidimensional, Cognitive impairment

## Abstract

**Background:**

Alzheimer’s disease (AD) leading to cognitive decline and dementia results from the interplay of multiple interacting dysfunctional biological systems. These systems can be categorized by domain, such as inflammation, cardiovascular health, proteostasis, or metabolism. Specific causes of AD differ between individuals, but each individual is likely to have causes stemming from multiple domains. Personalized multidomain therapy has been proposed as a standard of care for AD.

**Objectives:**

We sought to enumerate and describe prospective randomized controlled trials (RCTs) for multidomain interventions for AD, and to extract their inclusion criteria, trial design parameters (length, number of participants), and outcome measures. We sought to clarify gaps and opportunities in research and clinical translation.

**Methods:**

We conducted a scoping review using the standardized PRISMA-ScR methodological framework.

**Eligibility criteria:**

We include all cohort studies and RCTs for multidomain (also known as multimodal, multicomponent, multidimensional, or multisystem) therapy of any stage of AD, published for all dates through July 28, 2025.

**Result:**

There have been 23 studies (completed or reported as ongoing) of multidomain interventions for AD, including 19 RCTs. Of the 15 completed RCTs, 12 demonstrate benefit from their intervention in at least one arm.

**Conclusions:**

Although these RCTs differ widely in their parameters, the majority support the use of multidomain therapy, and show effect sizes greater than reported for unimodal therapies, including pharmaceuticals. Multidomain therapy should be the standard of care for AD. Multidomain interventions (also known as treatments) should be employed widely, early, and first-line. Treatment or prevention is likely to be most effective at early, presymptomatic stages, but is worthwhile at all stages of disease. In order to influence multiple domains, multiple modes of therapy are likely necessary in all patients. Some individual modes, such as particular lifestyle interventions, may target multiple domains. Nevertheless, most patients will benefit from multiple modes of intervention (multimodal intervention) that together target multiple domains. Standard-of-care guidelines should explicitly include multidomain interventions. Future clinical trials must be designed to iteratively improve multidomain therapies. Payors should embrace reimbursement for effective multidomain intervention, including personalized coaching.

**Supplementary information:**

The online version contains supplementary material available at 10.1186/s13024-025-00912-2.

## Background

Alzheimer’s disease (AD) results from the interplay of multiple interacting dysfunctional biological systems, or “domains”—such as inflammation, proteostasis, or metabolism. The causes of AD may stem from multiple genetic, immunological, and environmental factors. As such, AD is considered a “complex disease”. Translational research has enabled remarkable progress over the last fifty years in developing and deploying therapies for complex diseases such as hypertension and type 2 diabetes. Complex diseases—including AD—typically require complex therapies to prevent and treat dysfunction across many biopsychosocial domains and scales.^1^ Each of these domains of dysfunction can be treated by one or more interventions (also known as treatments) targeting that domain [1]. Specific causes of AD differ between individuals. Personalized, multidomain therapies are needed to best prevent and treat AD.

Multidomain interventions can slow progression, ameliorate, halt, or even reverse the course of complex diseases. In this Perspective, we discuss real-world approaches for applying existing knowledge to clinical care, maximizing knowledge from clinical trials and coupling that research to actionable interventions. We focus on the design and implementation of multidomain interventions, how to deploy these in the real world, and how to learn from these using dynamic dense-data clinical trials. These trials include longitudinal measurement of thousands of variables spanning many physiological domains [2]. We emphasize that lifestyle interventions, such as exercise and diet, target multiple domains; current evidence suggests they are the most effective treatment for AD [1]. We highlight remote and/or AI coaching as an approach for delivering complex interventions.

### Alzheimer’s disease and Alzheimer’s dementia

Cognitive impairment is decline in performance in one or more cognitive domains: learning and memory, language, executive function, complex attention, visuospatial, or social cognition [3]. Alzheimer’s disease (AD) is a category of cognitive decline that may lead to Alzheimer’s dementia. AD is a continuum that begins with the appearance of changes in the brain associated with the disease processes in asymptomatic individuals, which progresses through stages of increasing levels of disease-related brain changes, eventually leading to the appearance and progression of clinical symptoms. AD is diagnosed in individuals by assaying core biomarkers [4]. Individuals may have AD without being diagnosed. To have AD, an individual must be on a trajectory that would eventually lead to detectable AD biomarkers if they live long enough [5]. A divergence between the definitions of AD and Alzheimer’s dementia began to emerge in the late twentieth century and became more pronounced in the early twenty-first century [6, 7]. Alzheimer’s dementia is now defined as a very late stage of AD; these two “AD” acronyms may be conflated in some sources. Few authorities currently offer precise definitions of AD. Such definitions need to be useful both for researchers and for clinicians. It is difficult to come up with a definition suitable for both. Researchers generally desire narrow precise definitions that enable specific hypothesis testing and comparability between studies. Clinicians tend to favor broader definitions that enable diagnosis and treatment of patients in modern medical systems. However, there is some consensus bridging more clinical and more academic stakeholders: AD involves both cognitive decline and measurable pathology with alterations in AD-related molecular subsystems. AD was originally defined primarily as a clinicopathological entity in which the clinical symptoms of dementia were linked to specific amyloid plaque and neurofibrillary tangle neuropathological findings at autopsy [8]. However, over the first several decades of the twenty-first century, neuroimaging techniques and fluid (blood and cerebrospinal fluid) biomarkers have been increasingly successful at separating cognitive decline due to AD from other causes [9, 10]. In some cases, the molecular pathology of AD can be identified in individuals without any signs of cognitive decline [11]. The recent definition of AD published by the Alzheimer’s Association (AA) comes close to fulfilling the need for a precise research definition [4]. However, exact thresholds and recommendations or standardizations of biomarkers continue to evolve. A precise definition of AD remains elusive. Furthermore, the AA definition may neither fulfill the needs of clinicians nor capture the importance of tau pathology [12, 13].

Understanding the mechanistic or causal links between AD biomarkers, pathology, and symptoms is advancing steadily. Epistemic theories of causality of AD are rooted in the past in the sense that: (1) amyloid and tau biomarkers have been highly associated with disease, (2) genes encoding amyloid and tau pathways can cause AD, and (3) treatments targeting these pathways have some effects on symptoms. However, modern epistemic theories of causality recognize that these biomarkers may only be tangentially related, may not be required for causation, and that multiple other factors are also causal. Synaptic and neuronal loss results from multiple insults, including those resulting from upstream inflammation, lack of cardiovascular support, and metabolic dysfunction.

### AD has multiple causes

**Fig. 1:**
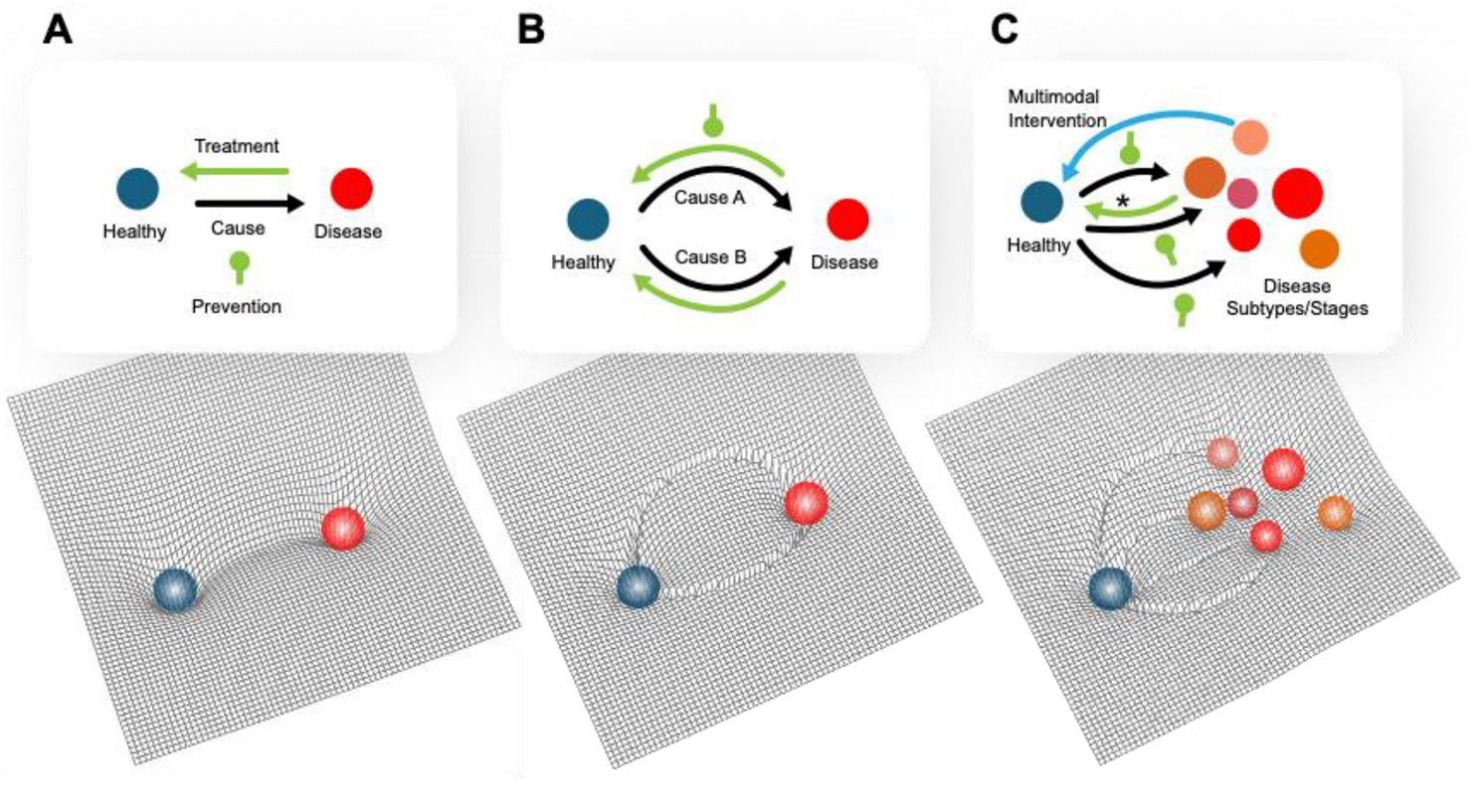
Conceptualization of an “energy landscape” for Alzheimer’s disease illustrating its nature as a complex disease requiring complex treatments. The *x* & *y* coordinates are shorthand for the millions of variables that represent the current status of the human biosystem. The slope of a path in the landscape can be considered the probability that an individual will move in that direction in a given unit of time. (**A**) *Classic concept of disease*: the system moves (black arrow) from a healthy state through a single obligatory causal state (e.g., head trauma) to a state of disease defined by clinical symptoms (e.g., chronic traumatic encephalopathy/CTE). Treatment consists of blocking (green club) or reversing the transition of the system along a linear path (green arrow). (**B**) *Extended classic concept of disease*: the system moves from a healthy state through one of several possible causal states (e.g., paraquat toxicity or GBA genetics) to a state of disease (e.g., parkinsonism). Treatment consists of blocking or reversing the transition of the system along one of the causal trajectories, as diagnosed in a particular individual. (**C**) *Complex disease*: the system moves from a healthy state through potentially multiple possible causal states to one or more similar but not identical subtypes, which in ensemble form a diagnostic category (e.g., Alzheimer’s disease), possibly transitioning or progressing between different states. Treatment consists of preventing the system moving towards unhealthy states, and/or moving the system towards the healthy attractor, possibly along different paths (blue arrow) than those used to reach the disease states (*). Multidomain therapies can work simultaneously to move the system along these favorable multidimensional trajectories

AD has multiple causes, but not all of these causes are known [14]. A *cause* of a disease is most strictly defined as *a specific factor without which the disease would not occu*r (e.g., [15]). This definition works well for diseases with simple linear chains of causation (Fig. 1A) and can even be adapted if there are multiple distinct possible causes converging on a single path (Fig. 1B). A more pragmatic definition of *cause* is *any factor that brings about change for better or worse in a health condition* [16, 17]. For purposes of conciseness and clinical relevance, we embrace this pragmatic definition, which encompasses both deterministic molecular pathways and modifiable risk factors (Fig. 1C). However—where possible—we distinguish between molecular causes and risk factors because (1) advances in precision & personalized medicine benefit from a mechanistic understanding of the relationships between risk factors and molecular pathways, and (2) modifiable risk factors vary in effect size and clinical importance whereas molecular pathways are deterministic. A reason to favor the more general definition of cause is that more than one deterministic molecular pathway may lead to AD. For example, it might be that either inflammation or amyloid accumulation or metabolic insufficiency alone could cause AD. Or—in many individuals—a combination of multiple molecular causes could drive AD onset and progression. Thus, it is important to avoid a paradigm that invites envisioning the cause of AD as ultimately confined to—or channeling into or through—a single path. With this conceptualization, it becomes easy to surmise that subgroups of AD arising from different molecular causes will likely require different treatments targeting different domains of intervention.

An alternative lexicographical viewpoint would be to define any clinical entity that cannot be understood as the result of single unbranched chain of causality not as a unique *disease*, but rather a *syndrome*. Such a syndrome, after sufficient future research, would eventually be reclassified as several separate diseases [18]. This may ultimately be true for AD, but we remain skeptical that resolving what we believe to be a web of factors that synergistically interact into separate definable diseases will ever be clinically practical. Furthermore, in practice, the momentum of historical usage of the phrase “Alzheimer’s disease” is so strong that it may never be renamed. Therefore, we are comfortable in our description of AD as a *disease with multiple causes*. Other complex diseases (or syndromes?) such as Parkinson’s disease (PD) face similar classification issues; Espay and Lang [19], for example, recognize that omics and other objective tests may assist in classifying subtypes of PD. They use a metaphor of duck attributes: every duck is unique but together they form a recognizable species (Fig. 1B from Espay and Lang).

The multiple causes of AD have been amply reviewed (e.g., [20–24]. There remain differences of emphasis by different stakeholders on whether and to what extent these causes merge onto a final common pathway (e.g., [25]). Different choices of definition—or even of biomarker threshold choices when employing the NIA-AA 2024 definition [4]—could lead to different lists of causes. Our approach to AD translational research is valuable for all proposed models.

### AD can be categorized into subtypes

Every individual with AD has their own set of causes of AD. In clinical practice, it is convenient to cluster groups of patients with a similar set of causes as a ‘subtype’ of AD (e.g., [26]); these individuals would occupy the same attractor state (or “well”) modeled in Fig. 1C. These states are more precisely defined by molecular sequelae (e.g., biomarkers) than causes, but the relationships between causes and sequelae (if measured with omics) should be approximately a bijection, so these perspectives are interchangeable. Systems approaches, in addition to *defining* AD subtypes, may be particularly useful in *identifying transitions*, or ‘tipping points’, into or between these subtypes (e.g., [27–29]). This systems view has implications for translational research and the design and refinement of AD therapies—whether lifestyle or pharmaceutical [30]. A therapeutic target (“drug target”) should not be a specific enzyme or molecule—or even a pathway—but rather should be *a network or a set of interacting networks*. This paradigmatic shift may justify greater conceptual emphasis on broad lifestyle interventions such as diet and exercise that may be highly effective but are better described as targeting networks than molecules. Each subtype may be modeled as an attractor state, and stages of disease may be modeled as a series of attractor states linked by transitional states (Fig. 1). Taherian Fard and Ragan [31] illustrate a method for quantifying the “energy” of such a landscape based on correlations of omics measurements and provide an example for PD. Statistical learning from omics data informs unbiased clustering of AD cases into subtypes [32–34]; such subtypes likely correspond to attractor states. Tijms et al. ascribe potential causes to five subtypes based on enrichment of analyte function metadata: hyperplasticity, innate immune activation, RNA dysregulation, choroid plexus dysfunction, and blood–brain barrier dysfunction; these likely correspond to a different mix of causes for cases in each cluster. Individuals with different risk factors are likely to be enriched in different molecular clusters corresponding to the causes of their AD. For example, specific genotypes—such as APOE4 homozygosity—may drive individuals towards particular attractor states [35, 36].

### AD can be categorized into stages

Somewhat orthogonally to the concept of clustering cases by molecular causes, one may cluster cases by the stage of disease. A given individual with the same set of causes will likely transition through multiple stages over the course of their disease. If these stages are sufficiently distinct, they may be defined by attractor states (Fig. 1C); if not, progression occurs smoothly along subtle contours of the modeled topography, and “staging” represents arbitrary but convenient thresholds. Historically—and to-date the most practical—methods of AD staging are based on cognitive assessment. A patient with AD but no symptoms is *presymptomatic*. A patient with complaints (perhaps brain fog, amnestic complaints, or executive function complaints) but who still scores normally on neuropsychological tests has *early AD* (and perhaps labeled with the deprecated term “subjective cognitive impairment”, or SCI due to AD). A patient with abnormal neuropsychological testing with preserved activities of daily living (ADLs) has *mid-stage AD* (and perhaps labeled with the deprecated term “mild cognitive impairment”, or MCI due to AD—even though there may be severe molecular dysfunction at such a stage). A patient with Alzheimer’s dementia has *late-stage AD*. Use of this historical approach to staging impairs consistency (not only between providers but also for the same provider across decades), as thresholds separating the stages are subjective and/or determined by the type and sensitivity of neuropsychological test(s). FAST staging [37] is one such approach: stage 1 reflects presymptomatic disease, stage 2 reflects SCI due to AD, stages 3 reflects MCI due to AD, and stages 4–7 reflect increasingly severe dementia. The CDR [38] provides a similar framework but lumps early stages (presymptomatic, SCI due to AD, and MCI due to AD) together in stage 0 (with SCI as stage 0 and MCI as stage 0.5), so is less useful. The CDR's resolution can be improved by utilizing “sum-of-boxes” to get the CDR-SB [39]. Improved molecular, imaging, and neurocognitive tests should soon enable more precise and consistent staging, particularly in presymptomatic individuals. One area of future research is to clarify whether stages represent distinct attractor states, or whether they are arbitrary designations based on thresholds of a continuum.

### Each of the molecular causes of AD may be driven by multiple modifiable risk factors

Proposed molecular drivers of AD include accumulation of amyloid β protein (Aβ) in neuritic plaques—or, more generally, disrupted proteostasis, disruption of the astrocyte-neuron lactate shuttle, and many other mechanisms (e.g., [40, 41]). Cardiovascular and metabolic dysfunction are two examples of modifiable causes of AD at a physiological systems level. These systems-level causes may each influence one or more specific molecular causes. Together, these encompass multiscale networks that influence molecular dysfunction of neurons and their synapses, and drive AD. Diet and exercise *both* influence *multiple* molecular system causes. Lifestyle and pharmaceutical interventions can be roughly categorized into domains that are generally more descriptive of the intervention (e.g., diet) than the physiologic and molecular systems they target. An oversimplification would be that each intervention domain corresponds 1:1 to a physiologic or molecular domain, but that is unlikely to be the case for most lifestyle interventions.

### Muti-domain or -modal?

We use the concept of “domain” to refer to categories of molecular subsystems, such as inflammation, proteostasis, cardiovascular, or metabolism. Therefore, a multidomain intervention is one that targets multiple subsystems (aka, “pathways”). We use the concept of “mode” to refer to a particular intervention, such as a particular pharmaceutical or diet. Most multimodal interventions are also multidomain interventions, but not necessarily, as multiple modes could conceivably all target the same domain. A mode of intervention (where “mode” is a descriptive classification of the type of intervention—such as “dietary”) could impact multiple molecular system domains. For example, exercise targets multiple domains. For reasons of conciseness, even though trials using only exercise as an intervention are therefore formally “multidomain”, we do not thoroughly review them here (e.g., they are not included in Table 1). “Multimodal” (or sometimes “multicomponent”) is used to indicate multiple types of intervention, even if they are targeted to the same domain [42]. ‘Multimodal’ and ‘multidomain’ are often confused, and in the literature are generally conflatable, along with ‘multidimensional’, ‘multisystem’, and ‘multi-domain’. None of these terms is synonymous with “lifestyle intervention”; a multidomain or multimodal intervention could consist entirely of pharmaceuticals, entirely of lifestyle interventions, or a mix of both. Current frameworks for quantifying the impact of particular modes of intervention (e.g., moderate aerobic exercise or coaching support for a Mediterranean diet) on particular molecular domains are insufficient [43]. Indeed, a precise delineation of each domain is also currently insufficient. Using omics together with other objective measures (e.g., imaging or neurocognitive markers) to better define domains and to measure changes in these domains in response to modes of intervention is a current research priority. We also recognize that some subfields use “mode” terminology to differentiate broader categories of intervention; notably in the context of exercise science, “mode” may refer to a particular type of exercise performed at a particular intensity. Our use is consistent with that terminology, but we would most likely not consider an intervention consisting solely of multiple modes of exercise to be multidomain.

We therefore undertook a scoping review. Our objective was to systematically map and describe all studies of multidomain interventions for AD, including cohort studies and prospective randomized controlled trials (RCTs), summarize their inclusion criteria, trial design, and reported outcome measures, and identify gaps and opportunities for research and clinical translation. We followed the PRISMA-ScR methodological framework, with Methods detailed in Supplemental Appendix 2. No formal quantitative synthesis or meta-analysis was performed, in line with scoping review methodology.

## Results

### Patients with AD need multiple interventions

Norton et al. [44], identified seven modifiable risk factors (which are also “causes” by the definition we are using in the present context) accounting for 30% of AD cases: low education, midlife hypertension, midlife obesity, diabetes, physical inactivity, smoking, and depression. In 2020, the Lancet Commission expanded these to twelve modifiable risk factors accounting for 40% of AD cases, adding: hearing impairment, low social contact, excessive alcohol consumption, traumatic brain injury, and air pollution [45]. Additional modifiable causes continue to be identified, such as untreated vision loss and high mid-life cholesterol [46]. The emergence of multidomain interventions for AD derived in large part from this evidence of modifiable risk factors.

Since AD has multiple etiologies, it makes sense that treatment—whether preventive, symptomatic, or curative—should target multiple domains. The best treatment is likely to be synergistic [47]. Molecular systems are highly intertwined and synergistic; improving function in one domain may improve function in other domains. Efforts to treat AD via a single domain—consider donepezil—have occasionally transiently and marginally improved function (e.g., [48]) but none have had the impact of multidomain interventions (Table 2). Most studies of multidomain interventions for AD show a significant and substantial effect on cognition. Of the 15 completed trials, only 3 (THISCE, JMINT, and AgeWell.de) do not show significant beneficial results, although three additional trials (Bae, LIEAD, & MAPT) with multiple arms or primary outcome variables did not show significance in all of these.

### The “placebo” effect

AgeWell.de, MYB, COMBAT, and US POINTER showed improvements in both the intervention and the control arm (Table 2) suggesting that the controls received substantial lifestyle benefit during the trial period and may have succeeded in ameliorating AD on their own. Such an effect in the context of a pharmaceutical trial might be considered a placebo effect, but it is likely that individuals who enroll in lifestyle intervention trials will change their lifestyle regardless of what arm they are assigned to or the intention of the trialists to partition intervention. Even simple interactions with trial staff may provide a modicum of “social” intervention. Control participants may be motivated to exercise on their own or pursue dietary health. Enrollment in a trial may reward altruism with improvements in mood. Such effects are likely to dampen significances and effect sizes in real-world multidomain trials compared to a gedanken trial with a theoretically perfect control arm, and likely explain improvements seen in some control arms. We conclude that many multidomain trials had less power than may have been expected. Addition of multi-omic measurements [49] to future trials will be critical to provide precise quantitative measurements of actual dose and exposure and effect on molecular systems received by all individuals in a trial (regardless of arm) to enable better interpretation of trial data and improved statistics. In the absence of a control arm with usual or standard care, trial designs should prioritize methodologies that facilitate within-subject comparisons, such as run-in or crossover designs [50, 51]. Selection of primary outcome variables should be based on their capacity to enable rigorous comparisons to reference cohorts.

### Controlled trials and cohort studies that inform multidomain interventions

Although some studies have looked at single interventions based on these risk factors (e.g., physical activity, cognitive training, or aggressive hypertension control), the results have not been compelling. The lackluster success of unimodal interventions was the impetus for studies of multimodal, multidomain interventions (Table 1), including the now well-known Finnish Geriatric Intervention Study to Prevent Cognitive Impairment and Disability (FINGER) trial in older adults at risk for cognitive decline [61]. We summarize multidomain RCTs for AD in a data extraction sheet in Table 1. Extraction of metadata for these trials is described in Supplemental Appendix 1. Related studies not meeting criteria for inclusion in Table 1 are summarized in Supplemental Appendix 2. FINGER showed that a 2-year multidomain intervention focused on diet, exercise, cognitive training, and management of cardiometabolic risk factors significantly improved neuropsychological functioning compared to a control group that received general education. The benefits of the intervention were seen irrespective of baseline factors including sex, age, education, socioeconomic status or cardiometabolic risk factors [77], although ethnic diversity was limited in this cohort. The success of FINGER has led to a global effort to expand on the findings, World-Wide FINGERS [78, 79]. A meta-analysis of 28 studies of multidomain interventions compared to single interventions in > 2700 patients with mild cognitive impairment (MCI) found that multidomain interventions resulted in significantly greater improvements in global cognition, executive function, memory, verbal fluency and other cognitive measures [80].

**Table 1:** Studies informing multidomain interventions

Study Name	Design	Domains	First Enrollment	Number of Enrolled Participants (P)	Length (months)	Baseline AD Stage (FAST)	Baseline AD Stage (NIA-AA)	Baseline Age Range (years)
MAPT^‡^	RCT	CDEV	2008	830‡	36	2	2	70+
FINGER	RCT	CDE	2009	1260	24	1–2	0–2	60–77
ENLIGHTEN	RCT	DE	2011	160	6	2–3	2–4	> 54
THISCE-EFF	RCT	CDE	2014	1082	12	1	0–1	> 64
CEDAR	cohort	CDEHSV	2015	174	18	1	0–1	adults (mean 60)
Aliabad-e Katul^‡^	RCT	CE	2017	22‡	2	4–5	4–5	60–85
Bae	RCT	CES	2017	83	6	3	3–4	> 64
COCOA	RCT	CDE	2018	55	24	3	3–4	58–89+
LIEAD	RCT	DEVS	2018	51	5	4	5	45–89+^†^
SMARRT	RCT	DE	2018	172	24	1	0–1	70–89
MYB	RCT	CDES	2018	6104	36	1	0–1	55–77
AgeWell.de	RCT	CDEHIS	2018	1030	24	1	0–1	60–77
COMBAT	RCT	CDES	2018	192	9	2	2–3	> 60
Xinzhuang	RCT	CE	2019	116	3	3	3–4	50–75
US POINTER	RCT	CDE	2019	2111	24	1	0–1	60–79
PREVENTION^*^	RCT	CDEV	2019	61	12	3	3–4	56–88
RECODE	cohort	CDEHISV	2019	25	9	3	3–4	50–76
Yang	RCT	CDE	2019	122	6	3	3–4	> 64
JMINT	RCT	CDE	2019	531	18	3	3–4	65–85
ITHNCLR	cohort	CDEHISV	2020	34	6	3–4	4	45–89
LETHE^*^	RCT	CDES	2022	156	24	1	0–1	60–77^†^
Evanthea^*^	RCT	CDEHISV	2023	72†	9	3	3–4	45–76^†^
BioRAND^*^	cohort	CDEHIV	2025	71	12	1	0–1	> 24

Staging is reported as best estimate from reported trial inclusion criteria (Supplemental Appendix 1). Trials without established names/acronyms are referred to by their geographic location. References: AgeWell.de [52]; Aliabad-e Katul [53]; Bae [54]; Biorepository Study for Neurodegenerative Diseases (BioRAND) [55]; CEDAR [56, 57]; COCOA [2, 58]; COMBAT [59]; ENLIGHTEN [60]; Evanthea (ClinicalTrials.gov ID NCT05894954); FINGER [61]; ITHNCLR [62]; JMINT [63]; LETHE [64]; Lifestyle Intervention for Early Alzheimer’s Disease (LIEAD) [65]; MAPT [66, 67]; MYB [68]; PREVENTION [69]; RECODE [70] ClinicalTrials.gov ID NCT03883633); SMARRT [71]; THISCE-EFF [72]; US POINTER [73, 74]; Xinzhuang [75]; Yang [76]. *In progress/final results not yet published. †Planned. NR = not reported. Domains (for details see individual trial publications as these are approximate): C = cognitive training; D = diet/metabolism; E = exercise; H = endocrine; I = inflammatory/infectious; V = vitamins/supplements; T = toxin reduction; S = social/psych. ‡more than two arms; some arms included multidomain interventions

Although all of these trials have generally consistent results, most with substantial improvement for those receiving multidomain interventions, there are numerous differences between the trials and their results. Quantitative meta-analysis would be ill advised. Some RCTs focus recruitment on individuals with presymptomatic AD, typically by identifying “at risk” individuals or individuals with cognitive test scores slightly below average, but still “normal”. Other RCTs focus recruitment on early AD, with individuals with objectively low cognitive test scores, but typically no loss of daily living function. Many of these early AD individuals would under previous nomenclature and classification systems have been classified as having MCI due to AD [81, 82]. No studies have focused on individuals with late AD (Alzheimer’s dementia), but some late AD individuals have been included in some of the RCTs (notably LIEAD, Table 1), as inclusion criteria are typically based on test-score thresholds and not presence/absence of dementia. Although these studies have limitations, there is no obvious trend of reduction of benefit in presymptomatic compared to early AD, and since there is no absolute reason why individuals with dementia would be completely unable to receive multidomain interventions—they can exercise, they can change their diet, they can take medicines—we would expect by extrapolation that at least some benefits would accrue at all stages of AD. However—in our subjective experience as clinicians—it becomes nearly impossible to introduce meaningful intervention in late stages of dementia, emphasizing the desirability of early intervention. One criticism of multidomain treatments is the lack of data from patients with Alzheimer’s dementia (as opposed to earlier stages of AD). Indeed, any potential value of multidomain treatments—or any treatment—in patients with more advanced dementia needs to be tested in clinical trials—but not at the expense of denying this care currently to patients with presymptomatic or less advanced disease. The effectiveness of multidomain treatments for prevention and treatment of early-stage disease seems clear.

In Table 1, trial size (P) and length of trial are positively correlated, and both are negatively correlated with baseline FAST (Supplemental Figure 1 & 2). These relationships support the perhaps commonsense hypothesis that logistics for large trials are easier for early-stage than for late-stage AD, particularly with regard to cost-per-participant, ease of recruitment, and ease of long-term follow-up. No relationships are observed between outcome significance or strength and any of trial size, trial duration, or baseline FAST. One would be reluctant to draw conclusions from these observations of lack of relationships, as the data from the observed trials only thinly samples all possible trial implementations, but these observations may hint that all of these factors together contribute to trial power, and that stakeholders designing or evaluating trials should consider all of these factors together in power analyses rather than focus on P alone. It remains unproven that (1) large AD trials are more powered than small AD trials, (2) late-stage trials are more powered than early stage, or that (3) longer trials have more power. Assumptions that these three statements are true should therefore be used with caution during trial design.

In light of these considerations, trial designers must navigate between the Scylla of real-world clinical desirability of intervention at a pre-symptomatic stage and the Charybdis of funding and logistical need for a short trial with classical statistical power to see a strong, significant result for a pre-declared endpoint—which in the last century required shorter trials with highly impaired individuals more likely to significantly decline in a control arm. Modern Odysseus-like trial designers should navigate this strait by using omics-based trials to observe early molecular changes in presymptomatic AD, thereby focusing on individuals most likely to benefit, with the most effect size, in a short time, using massive data, but avoiding the expenses, logistics, and risks—and perhaps impossibility—of recruiting many AD patients for years-long trials. Trials may leverage large, shared pools of potential participants such as ALZ-NET [83], and/or could explore novel methods of fully remote recruitment. Furthermore, participant baseline attributes, degree of adherence/compliance with the intervention, treatment received by controls, and other methodologic factors may account for some differences between multidomain intervention results. Therefore, future research in multidomain interventions should be able to comprehensively measure the system of each participant (including those in controls) both at baseline and throughout the period of study. Measures of adherence/compliance should include both non-molecular [43] and molecular measures [84]. These trials should be modernized with dense data designs, as we argue below.

### Excuses not to act

Futility has been suggested as a roadblock to action. Reasons for inaction arise in many forms, including: (1) “People with dementia cannot exercise”; (2) “The level of proof is not sufficient”; (3) “Costs are not reimbursable”; (4) “I just want to take a pill” (5); “I don’t know exactly what the best diet is, or the best course of exercise, so I won’t even try a potentially suboptimal intervention.” (6) “I want to start with just one thing, then add other interventions if it does not work”. These crutches of inaction illustrate many barriers, none of which are insurmountable, by individual patients, caregivers, clinicians, and/or society.

Education will be key to overcoming these barriers. Education is needed at all levels: policymakers, providers, caregivers, and patients. Coaching, whether by AI or in person, is likely to be an effective form of personal education. The very nature of multidomain interventions makes them exceptionally flexible. If an intervention (e.g., a gym membership) is too expensive, find another (e.g., a walk around the block) that is less expensive. If good intervention choices cannot be easily identified, leverage human or AI coaching. Perfection is the enemy of the good; any healthy diet is likely to be better than none (e.g., [85]). There is not time to iterate over each possible single domain. Iterating interventions is good where possible, such as in hypertension control. But for AD, waiting months or years to see the effects of each intervention in isolation will take too long – the disease will progress, healthspan will be significantly shortened. From a clinician’s point of view, and therefore a patient’s, multidomain intervention must start at diagnosis (or possibly before, based on risk factors), and be based on best available evidence, even if the perfect intervention may remain uncertain.

### Need to personalize multidomain interventions

There are multiple causes of AD. Multiple interventions may ameliorate AD. Different individuals with AD will have different mixes of causes. Therefore, personalized treatment will be needed [86]. These concepts are illustrated in Fig. 1. One of the challenges in implementing multidomain interventions, and in analyzing and interpreting their outcomes, is the variability in intervention design, including intervention length, treatment “dose” (i.e., number and frequency of sessions), and types of interventions included. Even within a trial, exact details of the multidomain intervention will differ between individuals. For example, if a goal is to reduce blood pressure across all participants receiving a multidomain intervention, the exact drugs and lifestyle changes to reduce pressure should vary between individuals. Even for fixed-dose pharmaceuticals, compliance, bioavailability, and half-life may vary between individuals. To address the need for scientific consistency in the presence of real-world variability, Solomon et al. [87] have proposed a model for “Brain Health Services” which allows for a degree of standardization while still personalizing the intervention to an individual’s specific clinical needs and motivation to change lifestyle behaviors. Nutrition interventions for AD prevention or treatment may be considered “personalized medicine” because many nutrients target specific pathways that have been shown to be altered in the disease. For example, a Mediterranean-type diet provides higher intakes of nutrients such as omega-3 fatty acids (from cold water fish), B-vitamins which lower homocysteine levels (from green vegetables and whole grains), and altered patterns of dietary fat types which can influence blood levels of lipids and phospholipids related to AD risk. This has been the basis of several personalized nutrition approaches to reducing cognitive decline in individuals at increased genetic risk for AD or with early-stage cognitive decline [88, 89].

If our underlying and overarching hypotheses are true—that AD has multiple causes and is best treated with multiple interventions—then conclusions and clinical recommendations resulting from such trials are likely to be applicable to all individuals regardless of where they are on the AD spectrum. We may be able to extrapolate conclusions from such trials to many real-world patients. For example, perhaps with help of caregivers, even individuals with advanced dementia are capable of modifying their diet, most are capable of exercising, and pharmaceuticals can be optimized for all. Multidomain recommendations can be adapted to the specific circumstances of each individual. It is also possible to classify each mode of intervention as either “general” (applicable to many conditions of health or disease, such as exercise) or “AD-specific” (applicable only to AD or very similar disease, such as donanemab). Such classification may help in didactics, epistemics, developing guidelines, and allocation of responsibilities between providers.

## Conclusions

### Personalization requires measurement

Advances in science are often dependent on advances in instrumentation, or the ability to measure things. To advance understanding and treatment of a systems disease such as AD, advances in measuring systems are needed. Such advances have happened in recent years, and continue to accelerate, notably by bringing down the cost and increasing the scope of multiomics. Advances in imaging and molecular biomarkers are also transforming AD care and research (e.g., [90]). Key needs for future advances will include optimizing signal-to-noise for measurements of system function and tracking the “dose” of each domain intervention. Most if not all lifestyle interventions are not precisely defined or quantified. There are infinite personal variations to food choice, timing, and amount even for concepts with specific names such as “Mediterranean diet” [91]. This vagueness extends to cognitive training and exercise domains. Some domains, such as sleep, are reasonably quantifiable; we need to bring similar quantification to all domains.

### Research requires measurement

Iterative refinement is key not only to clinical care of individuals but also to design clinical studies of the complex interventions that must be central to research-based guidelines for treating complex diseases such as AD. Iterative refinement of these complex interventions—such as diet and exercise—cannot be made until their effects can be objectively measured at the physiologic and molecular levels. This will require understanding these systems and their networks sufficiently enough so that quantifiable objective biomarkers—likely blood based—can be used to measure the impact of each intervention in each person on each system. Until these measurements are in place, studies and clinical trials will remain incomparable (Table 2). Meta-analyses will remain out of reach; validation studies will not yield insights into future trial designs. The ensemble of these omic, imaging, psychometric, and other data can be termed “systems quantification”. In addition to the value to research, systems quantification will also guide therapy, as summarized above [47]. A key element of systems quantification-one that is easily in reach of modern studies—will be to quantify compliance of study participants both with global and with specific interventions. Understanding compliance will enable interactive improvements in personalized recommendations. Soldevila-Domenech et al. [43] have suggested merging multiple domains into a single-dimensional compliance metric. We believe that a single metric of compliance could be valuable as a global indicator of the logistical performance of the trial infrastructure and implementation. However, because interventions and responses are multidomain and individual, a vector of compliance values will be necessary in order to advance understanding. Conversion of this vector of values to a global value should vary from individual to individual based on the particular nature of that individual’s needs to maintain and/or return to homeostasis. Furthermore, we anticipate that the most useful metrics will be objective and quantifiable and tied closely to molecular mechanism (such as molecular biomarkers of physiologic processes). These are likely to be more tightly correlated to causal influence on outcome measures than factors such as number of visits to a provider or amount of time engaged with cognitive training, due to the large number of factors (e.g., attention, alertness, exact nature of the intervention, personal traits, and many others) that modify or influence these measures on health outcomes. For example, diet questionnaires are nearly useless for understanding the impact of a particular diet change on a patient’s metabolism, but quantifiable molecular markers may highlight key successes and opportunities for improvements. Improvements in multidomain interventions will remain glacial until these domains are standardized by quantifiable biomarkers. Quantifiable biomarkers need not be liquid – they could be cognitive, imaging, functional, physiological, or other. They also need not be single or simple. They can be derived from numerous individual measurements across multiple dimensions. But they must be connected to causality, mechanism, or function—or they might be judged solely on Bonferroni-corrected p-values and will be disregarded. Use of these biomarkers will improve epistemology and lead to identification of mechanisms and relative contributions in populations and individuals.

**Table 2:** Results of multidomain intervention RCTs (trials included in scoping review)

Study Name	Primary outcome measure	Trial Duration (mos)	Relative difference (%)	Effect size (Cohen’s d)	Significance (p-value)
MAPT arm 1	Composite Z-score	36	∞	0.09	0.047
MAPT arm 3	Composite Z-score	36	∞	0.08	0.090
FINGER	Composite Z-score	24	25*	0.13	0.03
ENLIGHTEN	Ranked composite	6	~21	0.40	0.012
THISCE-EFF	MOCA_adj_	12	∞	0.2	0.094
Bae	NCGG-FAT battery	6	∞	1.5	0.003†
COCOA	MPI score	24	60	3.2	0.0007
COCOA	FAST	24	38	1.8	0.03
LIEAD	ADAS‑cog	5	∞	1.2	0.053
LIEAD	CDR-SB	5	15	0.68	0.032
SMARRT	Composite Z-score	24	74	0.14	0.02
MYB	Composite Z-score	36	280*	0.18	0.001
AgeWell.de	Composite Z-score	24	19*	0.01	0.87
COMBAT	Composite Z-score	9	948*	0.40	0.026
Xinzhuang	MMSE	3	∞	0.28	0.003
US POINTER	Composite statistic	24	14*	3.8	0.008
Yang	MoCA	6	∞	1.6	0.022
JMINT	Composite Z-score	18	∞	0.09	0.23

These studies are sufficiently diverse in design (e.g., with respect to population, outcome measure, nature of control arm, length, and intervention) as to discourage meta-analysis. No trials share any primary outcome measure (as composite scores are bespoke to each trial). Relative difference is between arms at trial endpoint. P-values are raw reported for multidomain arm vs control arm. Cohen’s d values are best estimates from reported trial data. MOCA^adj^ is a Taiwanese version of MoCA including a one-point addition for education < 12 years. ∞ = improvement (or no change) in intervention compared to decline in control; *both arms improved with respect to baseline. †Only one of six NCGG-FAT cognitive measures was significantly different. ‡MAPT arm 1 included omega-3 supplementation with other domains of interventions; arm 3 did not

### Systems cannot be understood a single dimension at a time

The main design goal of most multidomain trials is to test a synergistic basket of interventions. For example, some trials have demonstrated a synergy between exercise and cognitive training (e.g., [92]). However, in certain individuals, one or more domains may be particularly important, either in synergistic context or in isolation. Currently the best evidence for ranking of domains comes from epidemiology (e.g., [46]) and suggests that population attributable fractions of dementia are distributed somewhat evenly across about a dozen domains throughout the lifespan. Midlife hearing loss and high HDL cholesterol are at the top of this list, emphasizing the importance of treating these conditions in appropriate individuals.

A recurring criticism of multimodal trials is that they should never be undertaken until all single components of the multimodal intervention have been previously tested individually in a portfolio of many unimodal intervention trials. However, this is not possible. If near-infinite resources, volunteers, and time were available—and if the number of possible interventions were finite—one could conduct a clinical trial on every intervention in isolation, then combine those that worked additively to achieve the optimal multidomain intervention. However, some interventions have infinite variations (e.g., [93]), including diet and exercise (considering all doses, frequencies, and modalities): multimodal trials are inevitable and necessary even with improving technology and trial design.

Furthermore, we expect interventions to be synergistic, not additive. Therefore, a combinatoric approach to a clinical trial portfolio is not possible. Notably—as we have previously discussed [84]—such a research portfolio would take centuries, cost too much, and require more participants than are recruitable. In practice, embracing such a portfolio would mean no sufficiently complex multimodal intervention would ever be tested.

We designed the COCOA and its PREVENTION trials to address these concerns (e.g., [69, 94]). Soon, it may be possible to test many thousands of hypotheses in a single clinical trial using modern high-throughput technologies and clever epistemology—COCOA can be considered a step in that direction. Indeed, the practice of designing trials that look at overarching “patterns” of diet or other behavioral interventions—such as the Dietary Approaches to Stop Hypertension (DASH) trial [95]—has gotten wider acceptance in recent years. Like DASH, COCOA is not specifically designed to be a parallel test of many individual components but a synergistic test of a multimodal intervention that differs in particulars between participants due to personalization of the intervention. Collection of dense data enables systems quantification. This systems quantification enables improvement of recommendations for multimodal interventions by identifying molecular mediators and/or biomarkers that reflect mechanisms that may identify and explain the effects of individual components of the interventions that are most beneficial in particular individuals [96, 97].

Given sufficient data, it may be possible to estimate the number of attractor states—both homeostatic and dyshomeostatic—present in an individual’s cognitive landscape. Analyzing the dynamics of transitions between these states can help identify the minimal set of variables and dimensions necessary to model cognitive trajectories. Integration of this computational insight with detailed mechanistic knowledge should help elucidate the number of involved domains and guide efficient therapies targeting the minimal number of modalities best suited to treat that individual.

### Dense data enables clinical trials to perceive a whole greater than the sum of parts

Investigation of a complex system by perturbation of a single variable at a time is slow and will never provide more than marginal understanding. Classical clinical trials yield only marginal distributions of outcomes. The knowledge they provide is akin to what can be learned from querying a laptop computer with an ohmmeter. Many drugs, such as lecanemab, will continue to be identified with such classic FDA-sanctioned approaches. Nevertheless, given that the best therapies for AD are likely to be multidomain, newer epistemologies will be required to understand them sufficiently to fully realize their potential. Although we agree with a recent editorial that more multimodal RCTs are needed [98], these RCTs need to be carefully designed and used in conjunction with studies that generate knowledge of mechanism.

### Primary outcome measures

No pair of RCTs listed in Table 2 share any primary outcome measure. On the one hand, this speaks to the robustness of the benefits observed from multidomain interventions, as most of the trials showed benefit. It also underscores the evolving nature of outcome measures, and the continued quest to find better measures. On the other hand, it makes it difficult to compare trials or to perform integrative analyses (e.g., validation or meta-analyses) based on these primary outcome measures. Many trials report secondary measures or multiomics datasets, which do enable such integrative analyses. Insofar as Cohen’s d is a valued measure of effect size, then increase in accuracy and precision of the outcome measure (e.g., MPI Score vs composite Z-scores) may increase Cohen’s d (because of the effect of measurement error, or standard deviation, on Cohen’s d), resulting in improved sense of importance of trial results (see also Supplemental Figures 2–4).

Among trial results (Table 2), there is considerable variation in the statistical parameters (Cohen’s d and p-value) that are dependent on the accuracy and precision of the primary outcome measure. The trial with the most precise outcome measure (MPI score for COCOA) has the most significance but does not necessarily show a clinical effect (e.g., relative difference) greater than the other trials. One advantage of composite scores, such as bespoke composite scores or the more broadly accepted Clinical Dementia Rating – Sum of Boxes (CDR-SB), is they capture many dimensions of cognition in a single score; although the significance of the results from trials that use these as primary outcome measures may be lower, these composite scores suggest broad benefits of multidomain interventions across numerous cognitive dimensions. Despite that consideration, we recommend for future trials precise and accurate primary outcome measures to maximize power. In most cases, one cognitive and one functional primary outcome measure are warranted [99]. We recommend assigning composite measures as secondary outcomes to enable insights into robustness of results without sacrificing power.

Trials may benefit from assigning different outcome measures to participants at different cognitive stages. Measures designed for people with more severe dementia may not be sensitive to measure changes in individuals early in the AD spectrum, and vice versa. People may lose (or gain) different areas of cognition as they progress through their cognitive trajectory. They may show no change in an outcome related to a cognitive domain they have already lost (or have yet to lose) but show marked changes in a currently affected cognitive domain [100–102]. An elegant approach to integrating such insights would be to use the cognitive trajectory across the multidimensional landscape as an outcome measure.

### Trial quality

Trials vary in quality; the epistemological value of a trial depends on its quality. Trial length may be important for interventions for cognitive decline, as transient benefits such as seen in unimodal donepezil trials are of little benefit (e.g., [103]). Responses of complex disease to therapy are often non-linear, so the length of the trail needs to be long enough to capture potentially sudden changes between attractor states. As long as the trial is long enough to capture these changes, longer trials may not add much additional value. However, until the molecular landscape of AD is better understood, we can but speculate as to the ideal trial length. With this caveat, we recommend that trials should probably be at least 12 months in length. Of the 23 studies surveyed in Table 1, 13 were at least 12 months. High “**N**”—where **N** refers to the quantity of independent data points fed to a statistical workflow—is also important. Classically, for trials with two timepoints and one measured outcome variable, **N** is the number of participants (**P**), but for more complex trials **N** is also a function of the number of timepoints (**T**) and the number of variables (**V**). Very roughly, **N** ~ P*T*V, subject to—on one hand—data points being independent—but on the other hand—the data points working together to test a single hypothesis or reveal new knowledge. Most of the studies in Table 1 had fairly high **N**, but Aliabad-e Katul is an exception. High **P** may be best attainable for remote trials; remote trials have the added benefit of including rural participants (e.g., as in the MYB trial). High quality trials should also have arms with interventions sufficiently distinct so that comparisons between the arms have the potential to enable updates to clinical guidelines. Very few of the trials in Table 1 had an arm that was truly free of intervention. Many of the two-arm trials gave similar interventions to both arms but with one arm receiving less intervention. One opportunity for future AD trials is to utilize trial designs withs arms and interventions better structured to enable clear conclusions. Such designs must be ethical (e.g., provide at least standard care to all participants) and have robust recruitment (e.g., by making participation in the trial attractive, possibly by increasing post-trial participation benefits). This may be possible with trial designs where participants serve as their own control (e.g., crossover) or with other modern types of trial design (e.g., adaptive, pragmatic, or platform). High quality trials should use a standardized outcome measure that is comparable across studies; few studies in Table 1 did so. There are many other aspects of high-quality trials [104], including scientific rigor, implementation of robust standardized protocols, and following FAIR principles [105]. Most trials in Table 1 followed most of these recommendations. Improvements could be made by universal inclusion of EQUATOR or similar guidelines and checklists [106]. In summary, most trials in Table 1 implement many aspects of high-quality trials, but none are perfect. Of the three trials that did not show benefit (THISCE, JMINT, and AgeWell.de), none were particularly high or low in quality. It is very difficult to implement high-quality trials without budgets that originate from either big pharmaceutical companies or from governments with national health-care systems. Pharma seldom has the interest to (1) invest in dense data, as these data may reveal surprising knowledge, or (2) investigate non-pharmaceutical interventions. Therefore, funding for future high-quality trials may depend on interested governments.

### Comparison to other complex diseases

A diagnosis of hypertension encompasses a set of causes similar in biopsychosocial multiscale multidimensional structure to those for cognitive decline. Essential hypertension accounts for the majority of cases of hypertension, analogous to AD accounting for the majority of cases of dementia. Individuals with essential hypertension often have different mixes of causal factors; essential hypertension is a multifactorial condition resulting from many genetic [107], environmental, and behavioral factors [108]. Individuals with essential hypertension follow personalized disease trajectories, often stuck in dyshomeostaic attractor states. These attractor states may arise from hardened arteries [109], neurogenic activation [110], chronic inflammation [111], or renal dysfunction [112]. Personalized multimodal multidomain treatment is the best therapy to ameliorate essential hypertension [113]. Most cases of hypertension are successfully treated presymptomatically, with a minority of patients advancing to morbid stages. Mechanistic multiscale understanding of essential hypertension is considerably more advanced than our current understanding of AD, and serves as a beacon. Similar analogies to AD can be made for other complex diseases, such as diabetes.

### Insights

Lifestyle treatments are likely to complement, not replace, pharmaceutical treatments. Both are likely to be part of many optimal personalized multidomain therapies. Current pharmaceutical options have uncertain evidence [114], limited effect duration, constrained target population, or side effects (e.g., [115]) and offer limited range of targetable domains (e.g., most focus on proteostasis and produce marginal clinically relevant benefit as unimodal interventions). This presents an opportunity to develop a wider range of pharmaceuticals that target a range of treatment modes, and that can be easily adopted into personalized multidomain treatments. This need for multimodal therapies drives a need for cheaper drugs (cheaper to find, test, & make), that can be used in combination with each other to affect multiple systems simultaneously, and that allow for personalization (high safety and high ability to predict safety of drug combinations). Each individual drug might be used in only a small percentage of AD cases.

Lifestyle treatments tend to benefit even individuals without disease, so lifestyle interventions should be the foundation of multidomain therapies. Most patients with dementia have mixed dementia [5], so may benefit not only from the beneficial effects of multidomain therapies on AD but also from their beneficial effects on other components of their health. Lifestyle interventions should be emphasized in current treatment for all individuals on the Alzheimer’s disease and related disorder (ADRD) spectrum. Together, the trials in Table 2 provide strong evidence to elevate the prominence of these recommendations in guidelines and provider training. Lifestyle recommendations are often buried as afterthoughts at the end of multipage clinical recommendations that otherwise focus on pharmaceuticals (e.g., [116]). Lifestyle recommendations often lack specifics, making them hard to implement, particularly for practitioners who are not experts. Strong recommendations for multimodal lifestyle intervention should be placed before pharmaceutical recommendations in professional clinical guidelines and given equal or greater amounts of text. More research—some of which is ongoing, such as WW-FINGERS [78]—is necessary to make this case even more convincing [117]. The recently published U.S. Study to Protect Brain Health Through Lifestyle Intervention to Reduce Risk (U.S. POINTER) results also make this case [73]. More funding for lifestyle interventions should be applied both to research and via payors for medical care. The PREVENTION trial is nearing completion and should serve to iterate, validate, refine, and extend conclusions from COCOA and further test the methodologies discussed here [69]. Finally, pathways to *treat* ADRD may be different than pathways that *cause* ADRD (Fig. 1C). Research should be pursued to identify methods of perturbing systems to benefit AD patients, even if the “opposite” of those system perturbations would not cause AD. Hysteresis is a common property of systems, and there may be multiple paths into and out of particular attractor states; not all of these paths are reversible.

Understanding of the pathologies that lead to Alzheimer’s dementia is improving but incomplete. However, it is increasingly clear that the number of involved molecular domains and the severity of their involvement increases over time; treatment should begin as early as possible in AD [118]. In particular, those at the highest risk—for example, based on genetic screening—should initiate preventive treatment as early as possible. In all cases, treatment should start before the onset of dementia. We conclude that AD should be treated at its earliest stages, ideally presymptomatic—consistent with recommendations by other groups [5]. Individuals should be screened for risk, and those at highest risk should prioritize early intervention. However, if the early treatment window is missed, later treatment should not be denied. For example, patients who never receive diet intervention in early AD are still likely to benefit from diet intervention at later stages, but perhaps with less effect and requiring more assistance. AD interventions are likely to have at least some effect at all stages of disease, but we recognize AD as a continuum with on-going recruitment of additional disease perturbed biological networks, so interventions that are effective in early stages of AD are likely to be less effective in later stages. As the disease progresses, the specifics of certain interventions (like exercise) might have to be modified (e.g., for exercise: type, duration, frequency, setting, intensity, amount of caregiver assistance, required cognitive load), but the underlying value of targeting the molecular pathways influenced by these interventions is likely to remain useful.

### A plea for larger trials is hollow

Many papers end with an entreaty for larger and longer clinical trials. The motivation for such entreaties is that large trials are needed to generate small p-values and greater significance. The downside of such entreaties is that large trials that address all epistemological needs for AD translational research are either absolutely impossible (neither enough enrollable patients nor time in the universe) or practically impossible (no political appetite or commercial incentive to fund such trials). Furthermore, heterogeneity in large trials may do more to decrease than to increase power. Certainly, large trials should be encouraged and advocated for, and can address vital questions, particularly in large health care settings, such as whether particular policy implementations statistically improve population outcomes and save health care system dollars. Even though much of the basic knowledge necessary to drive rational implementation of multidomain intervention policies is already known, more knowledge is necessary to refine and improve interventions and broaden access to multidomain care. Such knowledge will have to come mostly from modern analyses of smaller cohorts and trials, given the impossibilities of all but a few large trials.

### Limitations

The primary limitation of this scoping review—and a major limitation of AD research—is the lack of clear diagnostic criteria for AD, particularly in presymptomatic stages. There are many trials for prevention of cognitive decline in healthy individuals. Unless a preponderance of these “healthy” individuals has AD without symptoms, such trials would be excluded. However, few published studies on healthy individuals report the percentage of participants with AD without symptoms. This presumably would require either sophisticated population-level epidemiologic inferences and/or expensive molecular or imaging biomarkers, many of which were unreliable at the time these trials were designed. Therefore, inclusion or exclusion of trials at NIA-AA and FAST stages 0–1 may be error prone. It is likely that some of the participants enrolled in every trial we cite (except PREVENTION, which measured biomarkers) did not have AD. However, many if not most participants probably had AD: most trials excluded participants with known non-AD causes of cognitive decline, and AD is the cause of the majority of cases of cognitive decline leading to dementia. Particularly for trials enrolling participants at early stages of the spectrum of cognitive decline, our conclusions may be better described as relating to cognitive decline generally, rather than AD specifically.

Estimation of values not explicitly reported in published reports (including age at enrollment, FAST stage, and effect sizes) may be error prone. Trials not referenced in the literature or indexed in PubMed or ClinicalTrials.gov may have been missed from this compilation. Trials not including key search terms, such as “multimodal” or “multidomain” may have been missed.

### Recommended policy and clinical implications

We need to educate adults about options to prevent and treat cognitive decline and dementia. Public health policy should include elements to address modifiable risk factors for cognitive decline and ADRD. In clinical trials, better assessments for outcome measurements are needed, including those that precisely and accurately measure specific domains of cognition and function; imprecise aggregate measures should be reserved for secondary outcome measures. Systems quantification should be universally deployed in trials, and more widely utilized to guide personalized medicine and health. Personalized coaching in a variety of forms (e.g., individual, group, AI-assisted) will be valuable to many; payors should support such coaching. Very inexpensive population-level options for each domain should be developed and deployed using advances in remote medicine to increase the reach of care to all individuals. The American Academy of Neurology’s (AAN) Committee on Public Engagement position statement on national brain health plans [119] includes calls to (1) optimize brain health through integration of preventive care practices, and (2) include brain health checkups as part of the standard of care across the lifespan. Switzerland has such a national plan that incorporates many of these ideas and recognizes the utility and need for multidomain interventions delaying age-associated cognitive deterioration [120], alongside blood biomarkers for population screening, and including effective pharmaceuticals to decrease the incidence of cognitive impairment and dementia. All nations should follow this lead and implement national brain health plans.

Multidomain interventions are best tailored to the individual’s personal circumstances (including genetic, environmental, and sociocultural context). Therefore, the best way to advance knowledge for any complex disease is a research program spanning many studies, many groups, and many years. However, most simple clinical trial designs would not be part of this research program, as they would provide only a narrow glimpse of the systems landscape. Each human differs from all others across thousands if not millions or billions of parameters. Efforts to create uniformity at baseline by matching on a few—or even a few dozen—of these parameters, such as race or sex or education, have some value but at best reduce the risk of confounding by a little while decreasing diversity a lot. Not only does a decrease in diversity impair society, but it also limits the generalizability and validity of translatable knowledge resulting from a trial.

Differences between participants are a strength as well as a limitation of all clinical trials. Although analytical methods for systems biology vary widely—ranging from statistical learning to mechanistic network modeling—they all require diverse perturbations on diverse systems. Classic trials designed to test the marginal effect of a single variable emphasize constancy on all other variables (“everything else being equal”)—particularly those that could influence the outcome. Constancy avoids confounding—the possibility that causal influences on the outcome are not due to the manipulated variable of interest, but rather to other variable(s) that exhibit diversity either at baseline or subsequently. But—in the context of complex diseases—such trial design has always been a mirage. Everything else is not equal: humans have far more variability than can be measured, let alone be controlled or be held constant. Of this variability, sex is particularly important, as the causes of AD differ in strength between men and women, and the response to lifestyle interventions differ [121]. The nature of complex disease is such that many variables that affect the outcome are likely to be unanticipated. Therefore, it is best to measure the entire system of each individual, understand them deeply, and model their trajectories. Biological plausibility must anchor epistemology [30, 122]. This is hard—particularly with twentieth century analytical techniques—but must be achieved if we are to make substantial progress in understanding and treating complex diseases in this century. In the immediate future, we recommend AD clinical studies trials be based on observational deep data of ~ 1000 patients. Such studies will capture data from individuals transitioning through different subgroups and different stages of AD, maximizing their value for systems biological analysis.

In this context of maximizing diversity by broadening inclusion and exclusion criteria, there is also value in focusing on particular populations. These populations should include individuals: (1) likely to have AD—allowing efficient allocation of therapeutic resources, (2) at earlier stages of pathogenesis more likely to be interruptible or reversible than later stages [123], and (3) with early-stage AD with sufficient function to understand and respond to coaching to implement multidomain recommendations. To enable this, staging should be comparable across studies; introduction of specific thresholds assayed with standardized protocols will be needed to operationalize biomarker-based staging criteria such as those described in 2024 NIA-AA guidelines [4]. Furthermore, solely from a perspective of trial design—to have statistical power and to complete the trial in a practical timespan—researchers desire to see a large and significant effect of the intervention over a short period of time. In early stages of the AD spectrum, control trajectories are likely to decline very slowly if at all, and a very large and/or long trial might be required. In dementia stages, individuals may be too impaired to respond to telephonically coached multidomain interventions. Therefore, pilot trials should focus on individuals partially along the AD spectrum. Ultimately, a priority for policymakers should be to enable multidomain interventions that can be conducted very inexpensively and burden free, so that they can be targeted at early stages of disease to entire populations. Improved early detection and risk stratification should fine-tune and target these interventions.

## Electronic supplementary material

Below is the link to the electronic supplementary material.

Supplementary material 1

Supplementary material 2

Supplementary material 3

Supplementary material 4

## Data Availability

No new data were generated for this manuscript.

## Footnotes

1 Abbreviations: AD, Alzheimer’s disease; ADRD, Alzheimer’s disease and related disorders (or nearly equivalently: Alzheimer’s disease and related dementias), DIAD, Dominantly inherited AD; FAST, Functional Assessment Screening Tool; MCI, mild cognitive impairment; MPI, Memory Performance Index; RCT, randomized controlled trial.
